# Point-of-care testing for cerebral edema types based on symmetric cancellation near-field coupling phase shift and support vector machine

**DOI:** 10.1186/s12938-023-01145-4

**Published:** 2023-08-15

**Authors:** Mingyan Li, Rui Zhu, Gen Li, Shengtong Yin, Lingxi Zeng, Zelin Bai, Jingbo Chen, Bin Jiang, Lihong Li, Yu Wu

**Affiliations:** 1 School of Pharmacy and Bioengineering, Chongqing University of Technology, Chongqing, 400054 China; 2 College of Artificial Intelligence, Chongqing University of Technology, Chongqing, 401135 China; 3 Department of Neurosurgery, Southwest Hospital, Army Medical University, Chongqing, 400038 China; 4 College of Biomedical Engineering, Army Medical University, Chongqing, 400038 China

**Keywords:** Cerebral edema types, Near-field coupling phase shift, Support vector machine, Symmetric cancellation

## Abstract

**Background:**

Cerebral edema is an extremely common secondary disease in post-stroke. Point-of-care testing for cerebral edema types has important clinical significance for the precise management to prevent poor prognosis. Nevertheless, there has not been a fully accepted bedside testing method for that.

**Methods:**

A symmetric cancellation near-field coupling phase shift (NFCPS) monitoring system is established based on the symmetry of the left and right hemispheres and the fact that unilateral lesions do not affect healthy hemispheres. For exploring the feasibility of this system to reflect the occurrence and development of cerebral edema, 13 rabbits divided into experimental group (*n* = 8) and control group (*n* = 5) were performed 24-h NFCPS continuous monitoring experiments. After time difference offset and feature band averaging processing, the changing trend of NFCPS at the stages dominated by cytotoxic edema (CE) and vasogenic edema (VE), respectively, was analyzed. Furthermore, the features under the different time windows were extracted. Then, a discriminative model of cerebral edema types based on support vector machines (SVM) was established and performance of multiple feature combinations was compared.

**Results:**

The NFCPS monitoring outcomes of experimental group endured focal ischemia modeling by thrombin injection show a trend of first decreasing and then increasing, reaching the lowest value of − 35.05° at the 6th hour. Those of control group do not display obvious upward or downward trend and only fluctuate around the initial value with an average change of − 0.12°. Furthermore, four features under the 1-h and 2-h time windows were extracted. Based on the discriminative model of cerebral edema types, the classification accuracy of 1-h window is higher than 90% and the specificity is close to 1, which is almost the same as the performance of the 2-h window.

**Conclusion:**

This study proves the feasibility of NFCPS technology combined with SVM to distinguish cerebral edema types in a short time, which is promised to become a new solution for immediate and precise management of dehydration therapy after ischemic stroke.

**Supplementary Information:**

The online version contains supplementary material available at 10.1186/s12938-023-01145-4.

## Background

Cerebral edema is an extremely common secondary disease in post-stroke. It is mainly divided into cytotoxic edema (CE) and vasogenic edema (VE). The former is the transfer of intracranial water from extracellular to intracellular. The latter gradually forms after the blood–brain barrier (BBB) is damaged, leading to the increase of intracranial water and the expansion of brain volume. It is easy to cause irreversible neurological damage and even form brain displacement and herniation [1]. So preventing the rapid development of VE is one of the keys in the neurosurgical monitoring field [2]. In addition, different types of cerebral edema need different treatment in clinical practice. For instance, the treatment of CE focuses on intracellular dehydration, while VE prefers to extracellular dehydration [3]. Therefore, the immediate diagnosis of cerebral edema type is very important for the precise management of stroke patients and the prevention of poor prognosis caused by the rapid formation and deterioration of vasogenic cerebral edema.

CT, MRI and other imaging methods can obtain high-resolution intracranial images in a non-invasive way, and identify the volume and location of edema with no pain to patients [4]. Minchew et al. have used MRI to evaluate brain edema 24 h after unilateral traumatic brain injury (TBI) in male and female rats [5]. In the same year, Fan et al. have measured the volume of postoperative brain edema on CT and MRI, and analyzed the correlation and consistency between the two. The results have shown significant comparability between CT and MRI in detecting postoperative brain edema [6]. However, such devices are bulky and generally need to be fixed, which makes it difficult to achieve real-time bedside monitoring of cerebral edema. The monitoring of intracranial pressure (ICP) can indirectly reflect the development of cerebral edema. However, the intervention of cerebrospinal fluid (CSF) and cerebral blood flow (CBF) compensation make ICP basically unchanged or less changed in the early stage of cerebral edema [7]. Transcranial Doppler (TCD) can monitor cerebral edema by measuring related indexes of CBF [8]. Fülesdi et al. have used TCD to evaluate the impact of changes in bleeding and edema volume on cerebral circulation, indicating that TCD monitoring can sensitively display hemodynamic changes caused by intracerebral hemorrhage [9]. Minchew et al. have used TCD to monitor a Chihuahua with acute cerebral infarction to evaluate local cerebral blood flow. They have proposed that TCD could be a non-invasive and easy-to-use bedside method for monitoring cerebral edema and infarction [5]. However, measurement results can be influenced by factors such as the acoustic window and probe position, which may compromise accuracy. Based on the changes in the absorption coefficient of intracranial contents to near infrared light, near infrared spectroscopy (NIRS) can achieve non-invasive monitoring of cerebral edema. Wang et al. have constructed a rat brain edema model using lipopolysaccharide. They have studied continuous non-destructive monitoring of ICP changes caused by brain edema based on near-infrared spectroscopy [10]. However, its penetrability is much weaker to human tissues, especially the skull, and the detection sensitivity of lesions in the deep position is significantly reduced [11]. Bioelectrical impedance can inject current from the skull surface to intracalvarium by electrode, and measure the changes of boundary potential to realize the non-invasive dynamic monitoring of cerebral edema [12]. Everitt et al. have demonstrated a novel intracranial bioimpedance monitor (BIM). It can distinguish between localized (such as bleeding) and global (such as edema) events [13]. But it is difficult to guarantee the electrical contact effect between electrode and scalp. The injection current attenuation and poor penetration caused by the high resistivity of the skull will also affect the measurement accuracy.

Bioelectromagnetic detection technology has many advantages, such as non-invasiveness, non-contact, good penetrability and real-time continuous monitoring. It has developed rapidly in the detection of brain diseases recently [14–17]. The theory of near-field coupled phase shift (NFCPS) originates from bioelectromagnetic detection technology. The alternating current of a certain frequency is used as an excitation signal, and generates an alternating main magnetic field through the excitation coil to pass through the measured target. Based on the near-field coupled effect, the current will be generated inside the measured target, forming a disturbance magnetic field relative to the alternating main magnetic field. There is a phase shift between the disturbance magnetic field and the alternating main magnetic field, namely, NFCPS. The earlier reported researches by Griffiths, Scharfetter and Gonzalez have indicated that the size of NFCPS is related to electrical conductivity of biological tissues [18–20]. In 2017, we established a real-time continuous cerebral edema monitoring system based on the frequency-dependent conductivity of biological tissues and the NFCPS theory. The 24-h monitoring experiments in rabbits prove its feasibility to detect VE [21, 22]. Combined with the two-port network test principle, we propose the frequency band selection method and improve the stability and sensibility of NFCPS in the detection of cerebral edema [23]. Meanwhile, a diagnostic model of the edema severity has been established by BP neural network. However, it is difficult to distinguish CE and VE by detecting NFCPS changes in the brain as a whole. The left and right hemispheres of the brain are approximately axisymmetric. In addition, the lesion hemisphere does not affect the healthy hemisphere in the early stage of cerebral edema [24]. Therefore, independent detection and cancellation of NFCPS in two hemispheres may enhance the difference information of CE and VE.

In this study, a symmetric cancellation NFCPS monitoring system is established. To explore the feasibility of this system to reflect the occurrence and development of cerebral edema, eight rabbits are performed 24-h NFCPS continuous monitoring experiments after focal ischemia caused by thrombin injection. After time difference cancellation and feature band averaging processing, the 24-h monitoring outcomes were analyzed and the corresponding features were extracted with the 1-h and 2-h time windows, respectively. Then, the classification models for CE and VE were constructed based on support vector machine (SVM) algorithm. To investigate the feasibility of using NFCPS and SVM to realize point-of-care testing for types of cerebral edema, the classification performance of multiple feature combinations in different time windows have been compared.

## Results

**Fig. 1:**
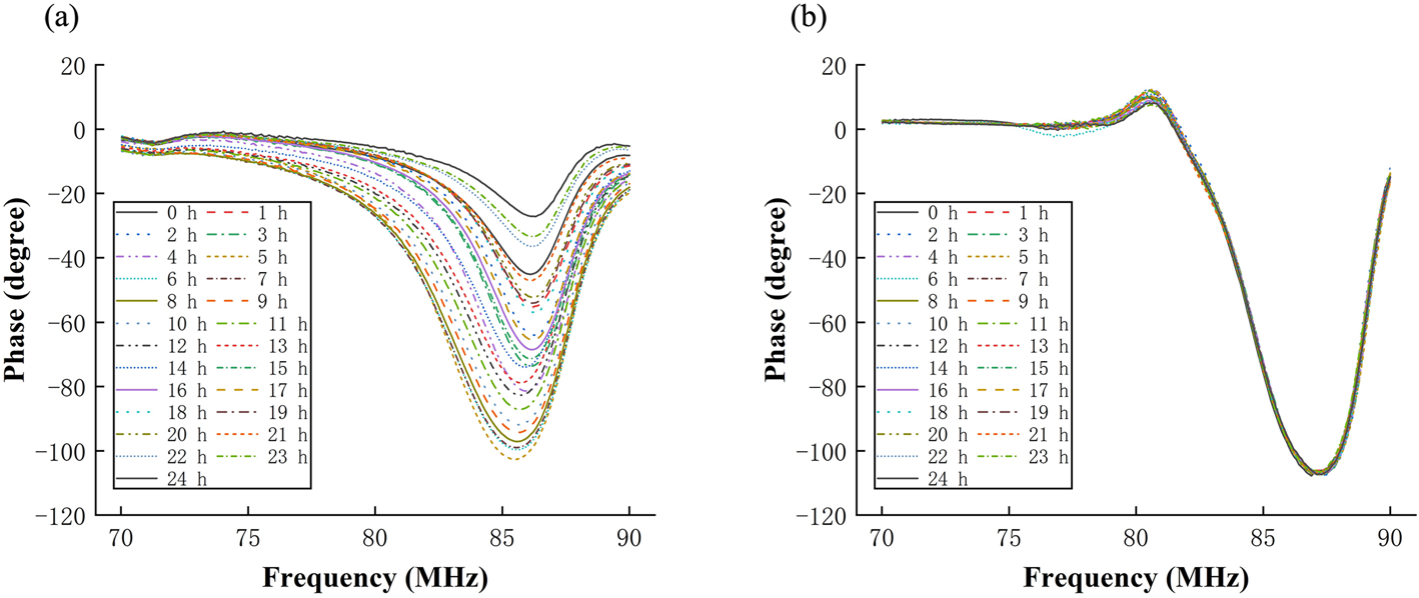
**a** The NFCPS of No. 8 rabbit in experimental group. **b** The NFCPS of No. 13 rabbit in control group

Figure 1a, b are the 24-h NFCPS monitoring outcomes in the detection frequency band (70–90 MHz) of No. 8 rabbit from the experimental group and the No. 13 rabbit from the control group, respectively. The NFCPS of No. 8 rabbit changes obviously over time, especially near the central frequency. The NFCPS of No. 13 rabbit displays no obvious change in the 24-h monitoring. The NFCPS in the detection frequency band at different times almost overlaps into a line. Since the rabbits in experimental and control groups undergo the same operation except for the injection of thrombin, the difference between them is due to the cerebral edema induced by focal ischemia. The results of the NFCPS monitoring for all rabbits are in Additional file 1.

**Fig. 2:**
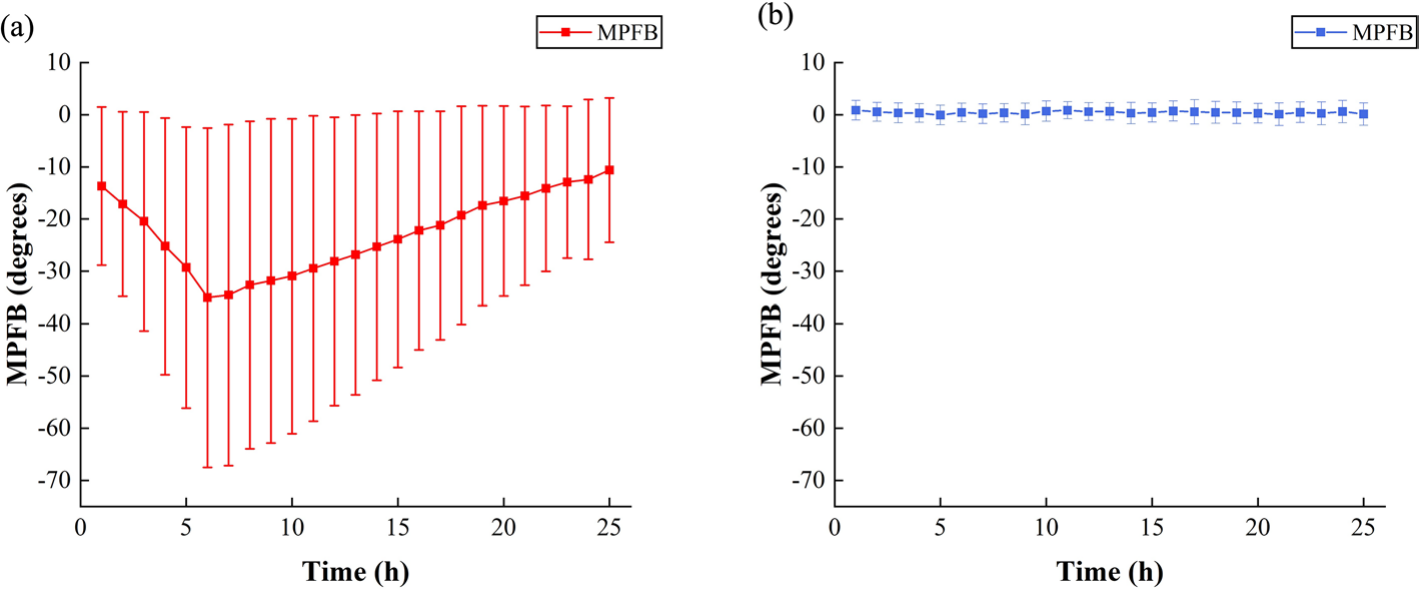
**a** The 24-h mean ± std of MPFB in experimental group (*n* = 8). **b** The 24-h mean ± STD of MPFB in control group

Figure 2a, b shows the change trends of mean phase in feature band (MPFB) in 24-h monitoring from the experimental group (*n* = 8) and the control group (*n* = 5). In the experimental group, MPFB shows a trend of first decreasing and then increasing, reaching the lowest point (− 35.05°) at the 6th hour. It is convinced that the change trends of MPFB in the experimental group have a relationship with the occurrence of CE and VE after cerebral ischemia. Song et al. have measured the electrical resistivity of the mice after focal cerebral ischemia injury, showing the trend of first increasing and then decreasing [25]. Schafer et al. have investigated the change trends in skeletal muscle resistivity due to ischemia and also obtained a trend of first increasing and then decreasing [26]. These results are the opposite of the trend in electrical conductivity. CE and VE play dominant roles successively after cerebral ischemia, showing a dynamic process [27]. The pathological process of ischemic stroke-induced cerebral edema theoretically causes the intracranial electrical conductivity to first decrease and then increase, which corresponds to the change trend of MPFB in the experimental group. Since the intracranial electrical conductivity of the rabbits in the control group has no dramatic change, there is no obvious upward or downward trend in MPFB. It only fluctuated around the initial value, with an average change of − 0.12° for 24 h. These results demonstrate that NFCPS technology can reflect the occurrence and development of CE and VE in post-ischemia.

According to the above analysis, the 6th hour in the experimental group was considered as the time point (*η*) of transition from CE to VE. Table 1 details the confidence intervals of MPFB for both the control and experimental groups at the 6th hour, as well as the *p*-values obtained from the independent samples *t*-test. Additionally, the table also displays the *p*-value from the paired samples *t*-test conducted within the experimental group between the 6th and the 24th hours. The *p*-values for these two time points are 0.046 and 0.018, respectively, both of which are less than 0.05, indicating a statistically significant difference. Concurrently, the calculation of confidence intervals demonstrates that the confidence intervals for the experimental group at these two time points are notably greater than those for the control group. Taken together, these results validate the efficacy of the NFCPS to detect normal status, CE and VE.

**Table 1:** The confidence intervals and *p*-value of MPFB in experimental group and control group

Samples	Test methods	Confidence intervals	*p*-values
6th hour control group	Independent samples *t*-test	[− 2.035, 2.874]	0.046
6th hour experimental group		[− 64.066, − 6.036]	
6th hour experimental group	Paired samples *t*-test	[− 64.066, − 6.036]	0.018
24th hour experimental group		[− 22.967, 1.756]	

**Fig. 3:**
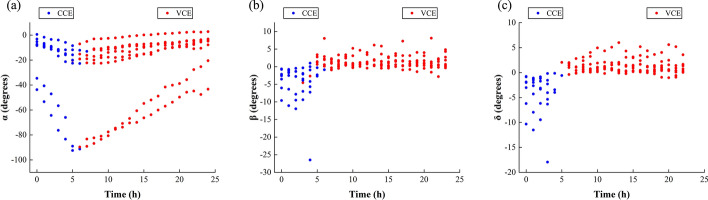
**a** Distribution of α during the 24-h monitoring process. **b** Distribution of *β* during the 24-h monitoring process. **c** Distribution of *δ* during the 24-h monitoring process

Figure 3a–c are the feature distributions of *α*, *β*, and *δ* during the 24-h monitoring process. The red points represent the VE-dominated stage, and the blue ones are the CE-dominated stage. The three features have a certain degree of discrimination for cerebral edema types, but there are some overlaps at the boundaries. The distribution of *γ* is almost identical to that of *β*, only shifted by 1 unit in the direction of the time axis.

**Fig. 4:**
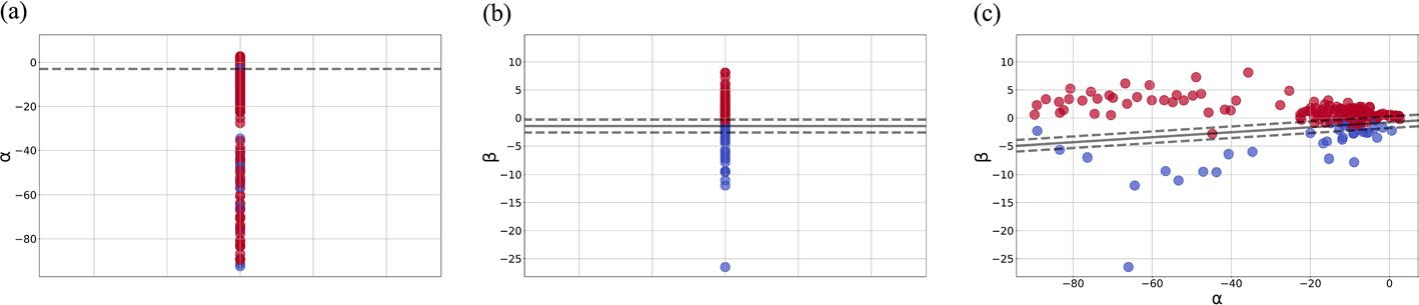
**a** Classification results using *α* alone. **b** Classification results using *β* alone. **c** Classification result of *α* and *β* together as a feature combination

The classification results of cerebral edema types under the 1-h time window are shown in Fig. 4a–c. The classification accuracy is shown in Table 1. When *α* (Additional file 2) is used as a training and identification feature alone, the linear kernel cannot find contour lines, and the classification accuracy is only 75%. MPFB generally shows a downward trend in the CE-dominated stage and an upward trend in the VE-dominated stage. Therefore, the classification performance using *β* (Additional file 3) is higher, and the accuracy reaches 92.31%. However, relying solely on *β* is easy to cause misdiagnosis when pathophysiological state of tested target is unstable. In order to enhance the classification robustness, we combined *α* and *β* as *αβ* (Additional file 4) for training, and the classification accuracy also exceeded 90% (91.89%). This feature combination can obtain the information about the change amplitude of MPFB relative to the initial value at *t*_0_ and the change direction next hour, which is helpful to reduce the misdiagnosis. The code of the SVM model is in Additional file 5.

**Fig. 5:**
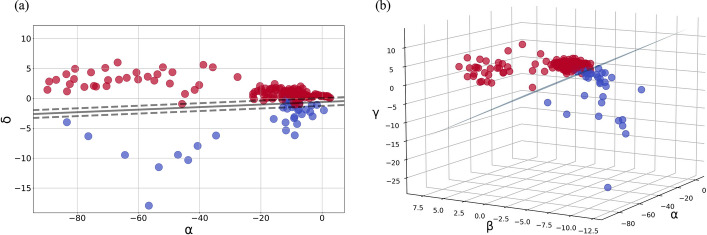
**a** Classification result of *α* and *δ* together as a feature combination. **b** Classification result of *α*, *β* and *γ* together as a feature combination

The classification results of cerebral edema types under the 2-h time window are shown in Fig. 5a, b. As shown in Table 2, the classification accuracy using *αδ* (Additional file 6) and *αβγ* (Additional file 7) is 97.06%, which is higher than that of the 1-h time window. It illustrates that increasing the monitoring time window can improve the classification accuracy.

**Table 2:** Classification accuracy in different time windows and feature combinations

Time slice	Characteristic parameter	Accuracy (%)^a^
*t*_0_–*t*_1_	*α*	75.00
	*β*	92.31
	*αβ*	91.89
*t*_0_–*t*_2_	*αδ*	97.06
	*αβγ*	97.06

^a^Accuracy takes two significant digits

**Fig. 6:**
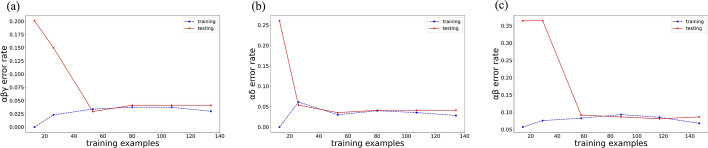
**a** Variation of error rates in the training and test sets under *αβγ* combination. **b** Variation of error rates in the training and test sets under *αδ* combination. **c** Variation of error rates in the training and test sets under *αβ* combination

As shown in Fig. 6, the training set and test set error rates under the three feature combinations tend to be equal with the increase of training samples. None of them exceed 0.05. This result illustrates that the classification model for cerebral edema types in this study based on SVM has no overfitting and underfitting.

**Fig. 7:**
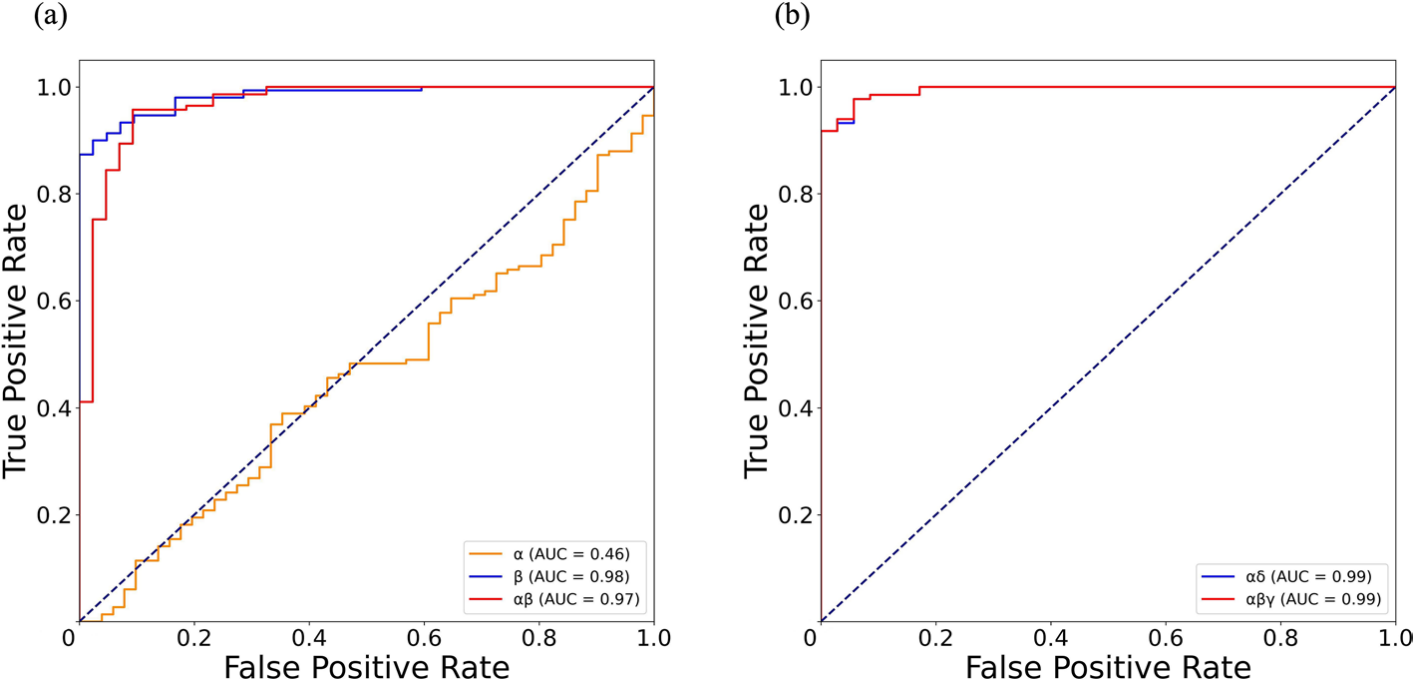
**a** The ROC curve of the features in the 1-h time window to differentiate CE and VE. **b** The ROC curve of the features in the 2-h time window to differentiate CE and VE

The receiver operating characteristic (ROC) curves of CE and VE classification based on the features extracted from the 1-h and 2-h time windows are shown in Fig. 7a, b. The area under the curve (AUC) of *αβ* as a classification training and recognition feature is only 0.02 lower than that of the two feature combinations under the 2-h time window (*αδ*, *αβγ*). It shows that classification specificity of the 1-h time window is almost equal to that of the 2-h time window. In other words, 1 more hour of monitoring has no obvious effect on improving classification specificity. Combined with the above performance on classification accuracy, it is found that CE and VE can be accurately diagnosed within 1 h based on NFCPS technology and SVM algorithm.

## Discussion

CE forms gradually with intracellular fluid accumulation in the early stage after ischemic stroke, resulting in brain parenchyma swelling [28]. At this time, the overall intracranial water content does not increase, and the blood–brain barrier is basically intact. With the further development of edema, the VE gradually predominates, resulting in the expansion of brain parenchyma volume, the increase of overall intracranial water content, and the intracranial hypertension [29]. Due to the lack of real-time bedside monitoring of cerebral edema, therapeutic intervention can only be performed after the development of VE to a certain extent, leaving a risk of delay. In this study, we propose a NFCPS method which can track the development and transformation from CE to VE in real-time. Combined with clinical targeted extracellular and extracellular dehydration treatment intervention, it is expected to establish new management guidelines and reduce the mortality and disability rate of ischemic stroke patients.

Compared with the traditional NFCPS detection methods, the symmetric cancellation NFCPS proposed on the basis of our previous study has superior performance. Due to the low electrical conductivity of biological tissue and the weak near-field coupling effect, the intensity of the generated disturbance magnetic field is much lower than the alternating main magnetic field, resulting in a weak NFCPS signal. Soleimani’s group have proposed multiple kinds of coil structures and detection methods to offset the main magnetic field [30]. However, due to the lack of detection on cerebrovascular diseases in vivo, the effect is not ideal. Gonzales et al. have developed the detection method of volume electromagnetic phase shift (VEPS) which can effectively weaken the interference of the main magnetic field and background noise, and rapidly obtain the instantaneous information reflecting the intracranial conductivity [31]. These works mainly enhance the detection sensitivity from engineering aspect, do not combine the characteristics of cerebrovascular diseases well, and have limitations in dealing with complex intracranial pathophysiological changes. Based on the symmetrical structure of the left and right hemispheres in the brain and the fact that unilateral hemorrhage does not affect the healthy hemisphere, Jin et al. design the hemisphere offset coil sensor [32]. The experimental results of intracranial hemorrhage in rabbits illustrate that its detection sensitivity is improved by an order of magnitude compared with the traditional coaxial coil. But the main magnetic field was canceled, making it difficult to measure the depth of the unilateral hemisphere. For this, we adopt a single excitation-dual detection coil sensor to independently measure and offset the NFCPS signals of the two hemisphere in a time-divisional mode with extremely short intervals, thereby enhancing unilateral lesion information without canceling the main magnetic field. Clinical observations have found that most unilateral stroke patients only produce edema around the lesion in the early stage, and do not immediately cause global brain edema [33]. Therefore, the symmetric cancellation NFCPS can eliminate the interference caused by other intracranial pathophysiological activities to a certain extent, so as to obtain the edema information more accurately. Besides, we combined the commonly used time difference offset method to filter out extracranial interference. In the previous study, we proposed the method to determine the optimal frequency of NFCPS detection for cerebral ischemia and edema based on the two-port network test principle [23, 34]. The animal experiments demonstrated that the sensitivity of NFCPS at this frequency was one order of magnitude higher than that of traditional detection method.

While the rabbit brain model used in our study cannot fully simulate the complexity and heterogeneity of human cerebral edema, it is widely recognized as a relatively ideal model animal, given the numerous physiological and anatomical similarities between rabbits and humans. From an anatomical perspective, the proportion of brain weight to body weight in rabbits aligns with that of humans. Similarly, the behavior of the blood–brain barrier in rabbits approximates that in humans, which is especially crucial for cerebral edema research since this barrier plays a central role in disease development. Compared to mouse brains, rabbit brains are larger and more complex, enabling us to gather more abundant information and achieve responses closer to those in human brains. In this study, we utilized a symmetric cancellation NFCPS monitoring system. Given that both rabbit and human brains possess an axial symmetric structure, the rabbit brain model can better leverage the advantages of this device. We firmly believe that the findings from this study will offer valuable insights into understanding human cerebral edema. Compared to measurements in rabbits, human measurements may yield larger disturbance signals and signals of interest. If appropriate data processing methods are employed, we speculate that the detection sensitivity of NFCPS will be further improved.

The 24-h continuous monitoring experiment of cerebral edema in rabbits illustrates that NFCPS can reflect the pathophysiological process of the CE and its gradual progression to VE. In this study, the rabbits in the experimental group were induced by thrombin injection to establish focal ischemia model. Compared with the common carotid artery ligation and epidural freezing method used in our previous studies, it is more consistent with the pathophysiological mechanism of cerebral edema induced by ischemic stroke [35]. Compared with the experimental group, NFCPS and MPFB in the control group have almost no change. The MPFB in the experimental group is first decreasing and then increasing during 24-h NFCPS monitoring, which is consistent with the pathophysiological process of ischemic stroke-induced CE and progressive development of VE. In the early stage of cerebral ischemia, CE allowed extracellular fluid to enter the intracellular space, resulting in cell swelling and reduced space. Therefore, the electrical conductivity of brain tissues decreased, resulting in the decrease of MPFB in the experimental group. With the continuous development of cerebral ischemia, the integrity of BBB was destroyed, VE gradually dominated and the perfusion of CBF and CSF increased in extracellular fluid [36]. The electrical conductivity increased in this stage, resulting in increased MPFB. Although the time of the transition from CE to VE in different rabbits was not the same, they all concentrated in the range from 5 to 7 h after modeling. More importantly, it is expected to judge the cerebral edema types based on the change trend of MPFB, which is meaningful for the targeted treatment of ischemic stroke-induced edema.

Based on the SVM and the features extracted from the continuous monitoring of NFCPS, CE and VE can be diagnosed immediately. SVM is a small sample machine learning algorithm. It can capture sample information and eliminate redundancy with good generalization ability, getting the global minimum value of the objective function [37]. The key to the SVM algorithm is the selection of the kernel function and the penalty factor. The data in this study are linearly separable, so a linear kernel with less main parameters and fast running speed is selected. In addition, the occurrence time of VE among different samples is not the same, and the classification model should possess a high generalization ability to predict unknown and fresh data. For this, a penalty factor ($C$) of soft-margin was introduced in this work. When $C$ tends to infinity, samples with classification errors are not allowed, and the probability of overfitting is very high, making it difficult to deal with individual differences of targets and increase the misdiagnosis rates. When $C$ tends to 0, the model no longer focuses on classification accuracy but on larger intervals [38]. This will affect convergence and lead to increased misdiagnosis rates. Combining with the variation of error rates, the parameters of classification model with the optimal performance are determined by K-fold cross-validation [39]. In the 2-h time window, two feature combinations (*αδ*, *αβγ*) that most directly reflect the classification performance were selected. There is no inherent difference in the classification performance between *β* and *δ*, so they are not used together as a feature combination. By analyzing the classification performance of different time windows, it is found that the accuracy of the 1-h time window reaches more than 90%, and that of the 2-h time window gets a little improvement. Compared with such a low performance improvement, it manifests important clinical significance to instantly identify cerebral edema types and take timely targeted intervention under the condition that the accuracy of the 1-h time window is sufficient. The classification accuracy of *α* and *β* together as a feature combination is a little lower than that of *β* alone in this work, the reason of which is that their random states are different. In parameter optimization, the random state corresponding to the median accuracy of *β* is 5, and that corresponding to *αβ* is 1. In other words, the difference in the distribution between the training and test sets is mapped onto the classification results. Considering that *αβ* reflects the change amplitude and slope of the NFCPS signal at the current detection time, they should be used as a feature combination to identify the cerebral edema types in practice.

### Limitations and next works

There are some limitations in this study. (i) The pathophysiological process of ischemic stroke-induced cerebral edema is complex. The occurrence and development of CE are often accompanied by VE. (ii) Averaging processing has limited effect on improving the poor consistency of NFCPS monitoring outcomes caused by individual differences. (iii) A comparative analysis of the performance on discriminating cerebral edema types by multiple machine algorithms has not been performed. In the following work, we will further explore the characteristics of NFCPS signal during the development of VE on the basis of increasing the sampling frequency, and carry out a comprehensive analysis of its weight in different stages after ischemic stroke. In addition, the convolution operation will be introduced into the NFCPS monitoring outcomes to extract features. Combined with the feature selection based on principal component analysis (PCA), the classification performance on classification of cerebral types by multiple machine learning algorithms (neural networks, ensemble learning, random forest, decision trees, et al.) will be compared to a more optimal model for early warning of VE. (iv) Limited sample size and animal model. In future studies, we will consider increasing the sample size to enhance the reliability of the research results. To improve the applicability of the measurement and monitoring methods used in this study, it is necessary to further conduct clinical experimental design work that aligns more closely with the realities of clinical practice, such as the design of cerebral edema models and data collection for various subtypes of cerebral edema. This will aid in uncovering the actual progression of cerebral edema, particularly the differences in signal changes between different stages.

## Conclusion

Based on the fact that the left and right hemispheres of the brain are approximately axisymmetric and unilateral local edema does not affect the healthy hemisphere, a symmetric cancellation NFCPS monitoring system for cerebral edema is constructed in this study. After symmetric cancellation, time difference offset, and calculation of mean phase in feature band (MPFB), the 24-h monitoring outcomes of cerebral edema in rabbits illustrate that NFCPS could reflect the occurrence and development of CE and VE in post-ischemic stroke. According to the 24-h MPFB trend of decreasing first and then increasing, we extracted the features (*α*, *β*, *γ* and *δ*) under the 1-h and 2-h time windows, respectively, and built a classification model of the two cerebral edema types combined with SVM. When *α* and *β* from the 1-h time window are used together as a feature combination, the classification accuracy is higher than 90% and the specificity is close to 1. This performance is almost the same as those in the 2-h time window, which proves the feasibility of distinguishing CE and VE in a short time by NFCPS technology and SVM algorithm. This study proposes a new solution for tracking the occurrence and development of cerebral edema after ischemic stroke and lays the foundation for developing the precisely personalized management of dehydration treatment.

## Methods and materials

### Detection principle

**Fig. 8:**
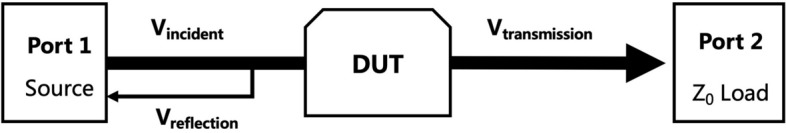
The schematic diagram of the two-port network

According to the two-port network theory, the brain can be seen as the device under test (DUT). Figure 8 is the schematic diagram of the two-port network. At a given frequency, the brain can be equivalent to a frequency-dependent complex impedance ($Z_{{\text{L}}} = R + j\omega X$). When *Z*_L_ is connected to the two-port network, its changes will modulate the transmission coefficient [40].

The transmission coefficient refers to the relationship between the transmission signal and the incident signal:

1 $$ {\text{Transmission}}\;{\text{coefficient}} = \frac{{V_{{{\text{transmission}}}} }}{{V_{{{\text{incident}}}} }} = \tau \angle \varphi , $$

where *τ* is the amplitude of transmission coefficient. *φ* is the phase of transmission coefficient. The size of *τ* determines the efficiency of transmission, affecting the detection sensitivity and stability. The magnitude of *φ* is related to the conductivity of DUT. When its conductivity is changed, the *φ* will shift, that is NFCPS. Regardless of the mechanisms of edema and accumulation of fluid, a change in the water composition of the tissue is bound to change the electrical conductivity of brain tissue.

### Symmetric cancellation NFCPS monitoring system

**Fig. 9:**
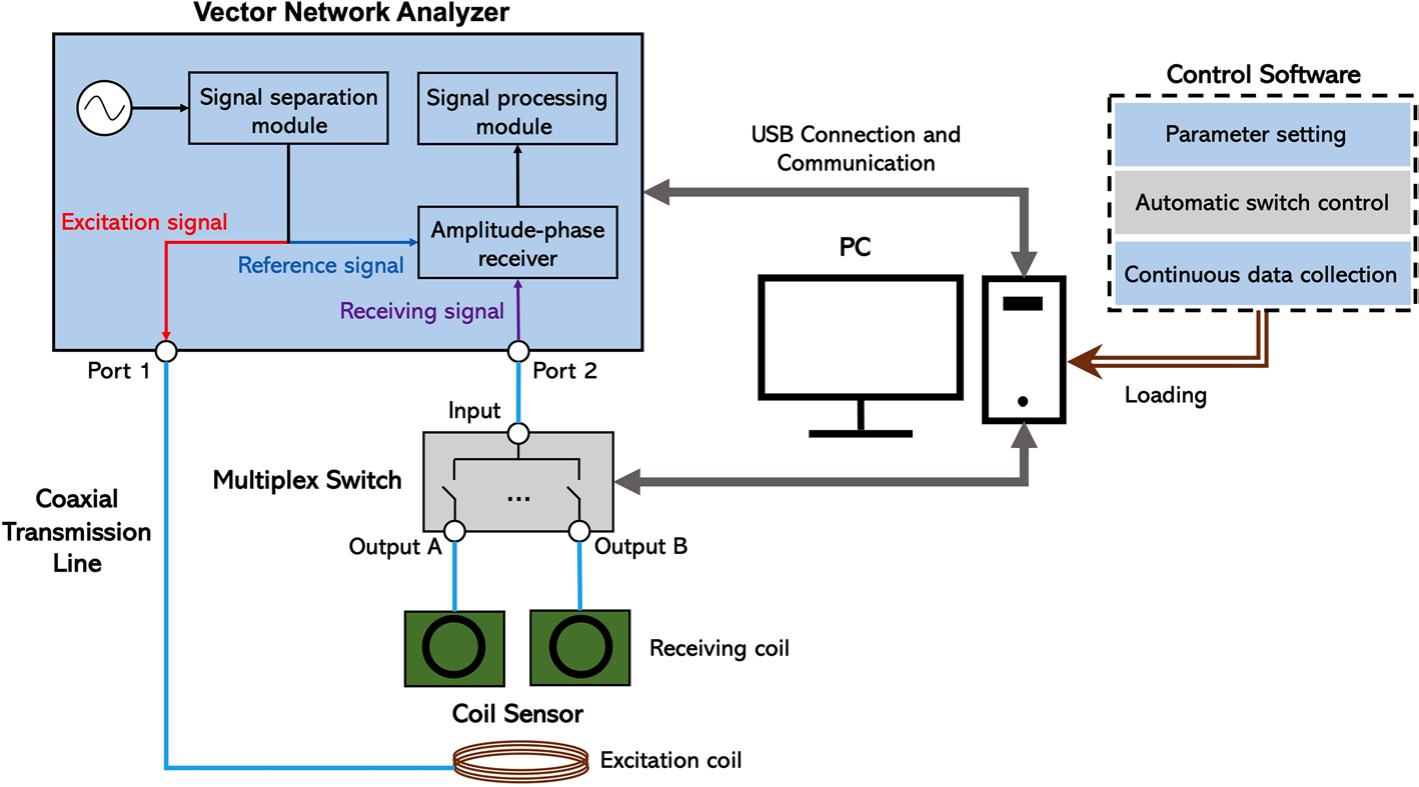
Construction of symmetric cancellation NFCPS monitoring system

As shown in Fig. 9, the symmetric cancellation NFCPS monitoring system consisted of coil sensor, portable vector network analyzer (Copper Mountain, M5065), multiplex switch, PC and control software.

**Fig. 10:**
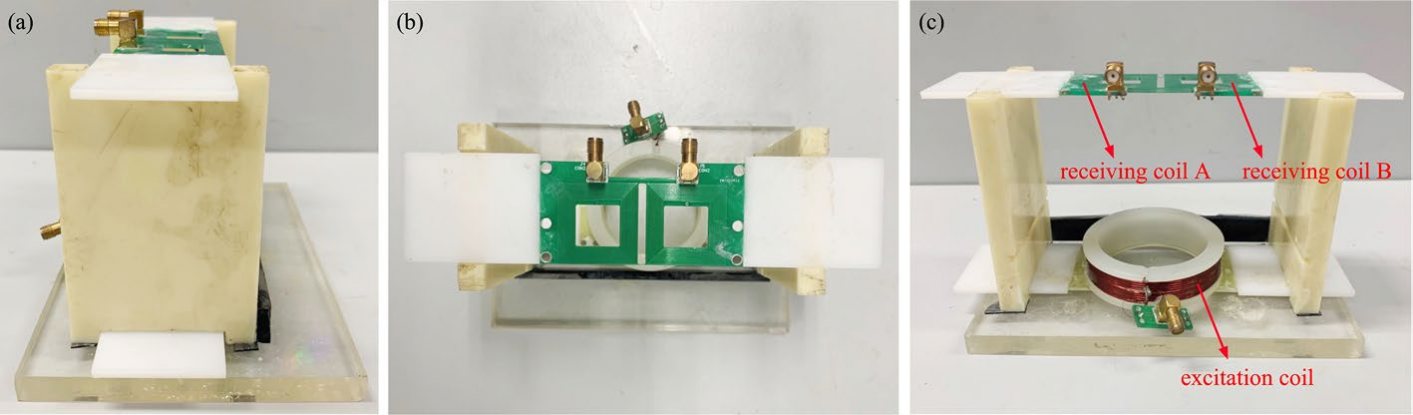
Single excitation-dual receiving coil sensor: **a** left view, **b** vertical view, **c** front view

The coil sensor consisted of an excitation coil and two receiving coils, and its main, left and top views are shown in Fig. 10. An acrylic cylinder with a diameter of 6.5 cm was wound by an enameled wire with a diameter of 0.8 mm to construct the excitation coil, and the number of wire turns was 10 turns. The receiving coil was wound 16 times by a wire with a diameter of 0.4 mm, forming a square with an outer side of 29.2 mm, an inner side of 17.2 mm, and a hollow in the middle. The excitation and receiving coils were fixed into a cube (length 13.5 cm, width 6 cm, height 11 cm) suitable for rabbit skull through plexiglass. The excitation coil can flexibly slide to adjust the symmetry. The measured brain was placed between the excitation coil and the receiving coil. One receiving coil was placed above the normal hemisphere, and the other was placed above the ischemic hemisphere. According to detection principle in 2.1, the two receiving coils formed two two-port networks with the excitation coil, respectively, to achieve independent detection of the healthy and ischemic hemispheres. Therefore, the difference between the two groups of detection results, namely the symmetric cancellation NFCPS, theoretically only reflected the lesion information.

The vector network analyzer (VNA) connected to PC by USB line was the hardware core of the entire system. It had two ports (Port 1 and Port 2), responsible for providing an excitation source and transmitting a near field to the measured target through Port 1. At the same time, the transmission signal was collected at Port 2. The NFCPS test outcomes were finally obtained after completing the signal processing and phase calculation.

There were two inputs and eight outputs in multiplexing switch. One input was connected with the Port 2 of the VNA, and two of the corresponding four outputs (Output A and Output B) were connected with receiving coils. The Port 1 of the VNA was directly connected with the excitation coil. The multiplexing switch can alternate rapidly at the output port connecting the two receiving coils to realize the synchronous acquisition of the two NFCPS signals.

The control software was responsible for communication with the multiplexing switch and the VNA to complete the setting of measurement parameter, target alternation of multiplexing switch and the continuous collection of NFCPS data. As mentioned in 2.1, the higher the transmission coefficient amplitude, the better the detection performance. The frequency band with higher amplitude of conditional transmission coefficient of this system was concentrated in 70–90 MHz. Therefore, it was used as the NFCPS detection frequency band, and other measurement parameters are shown in Table 3.

**Table 3:** Measurement parameters of NFCPS

Name of parameters	Setting
Sweeping points	201
IFBW	3 kHz
Output power	5 dBm
Trigger source	Internal
Trigger mode	Continuous
Sampling interval	1 h
Data format	Phase

### Twenty-four-hour monitoring of cerebral edema in rabbits

For evaluating the feasibility of the symmetric cancellation NFCPS to monitor the occurrence and development of cerebral edema, we carried out 24-h continuous monitoring experiments in rabbits. The experiments were approved by the Laboratory Animal Welfare and Ethics Committee of the Chongqing University of Technology. All the animal procedures complied with the ARRIVE guidelines and were carried out in accordance with the National Research Council’s Guide for the Care and Use of Laboratory Animals. We respected animal life during the experiment, taking measures to minimize animal stress, pain, and injury. After the completion of the experiment, euthanasia was performed. Thirteen healthy male New Zealand white rabbits (2.5 ± 0.5 kg) were provided by the Animal Center of Army Medical University (SYXK 20170002) and divided into experimental (*n* = 8) and control groups (*n* = 5) using a random number table method. All the rabbits were delivered to the animal laboratory 1 day in advance for feeding. Sufficient food and drinking water were provided, and suitable feeding conditions were set up. The room temperature was controlled at 22 °C, and the relative humidity was maintained around 50%. For the experimental group, the focal ischemia-induced cerebral edema model was established by thrombin injection [41, 42]. Rabbits were anesthetized by a urethane solution (SCR, Shanghai, China) with a volume fraction of 25%. It was intravenously injected through the rabbit ear rim at a dose of 5 mL/kg. Meanwhile, 0.2 mL/kg of atropine sulfate (0.5 mg/mL, ELIPEX, Shanghai, China) was intramuscularly injected. The bovine thrombin and rabbit brain prothrombin were diluted with normal saline, and the mixture was prepared in the ratio of 1:10. The fascia and muscle on the right side of the trachea were separated abruptly, and the right common carotid artery was exposed and extended to the head along the common carotid artery. The internal carotid artery and external carotid artery were dissected and exposed. Then, 3 mL of the mixture was injected into the deep internal carotid artery (25 μL/min) by microinjection pump.

**Fig. 11:**
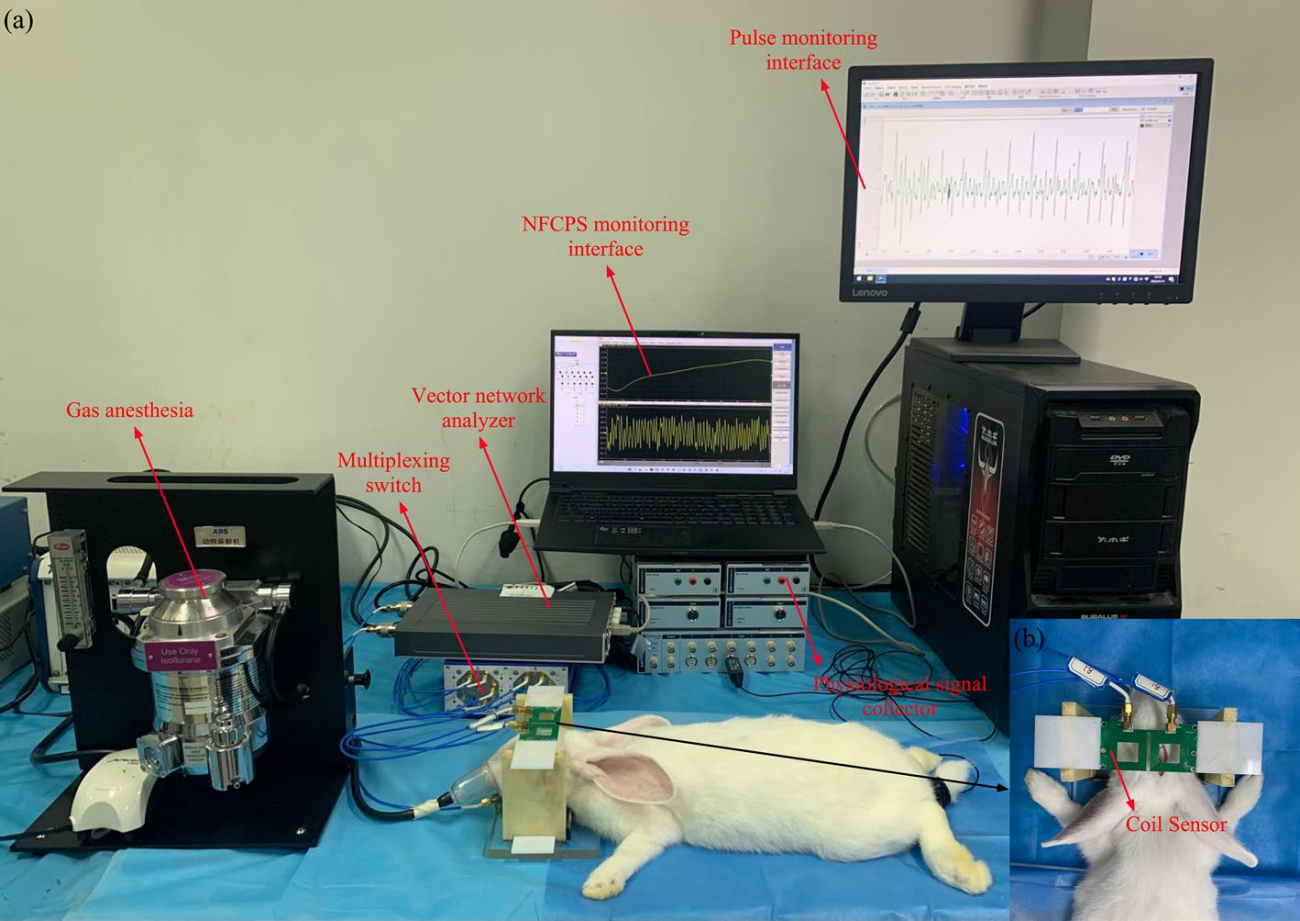
**a** The experimental platform of 24-h NFCPS monitoring in rabbits. **b** The relative position between the coil senor and the rabbit’s head

As shown in Fig. 11, the symmetric cancellation NFCPS monitoring system was used to monitor rabbits continuously for 24 h after modeling. The rabbit skull was placed between the excitation coil and receiving coil, and the sagittal suture was located in the middle of the rectangular opening (length 29.2 mm, width 0.3 mm) between the two receiving coils. Physiological signal collector was used to monitor rabbit’s pulse. NFCPS data of the left and right hemispheres were collected after the rabbit’s pulse was stable. During the monitoring process, rabbits were subjected to continuous anesthesia using 2% isoflurane gas at a flow rate of 0.6 L/min. Meanwhile, pulse frequency was monitored to ensure the vital sign stability. Rabbits were euthanized via IV pentobarbital overdose at the end of monitoring. Except for thrombin injection, the rabbits in the control group underwent the same operation as the experimental group, and was continuously monitored by 24-h NFCPS.

### Data process and analysis

**Fig. 12:**
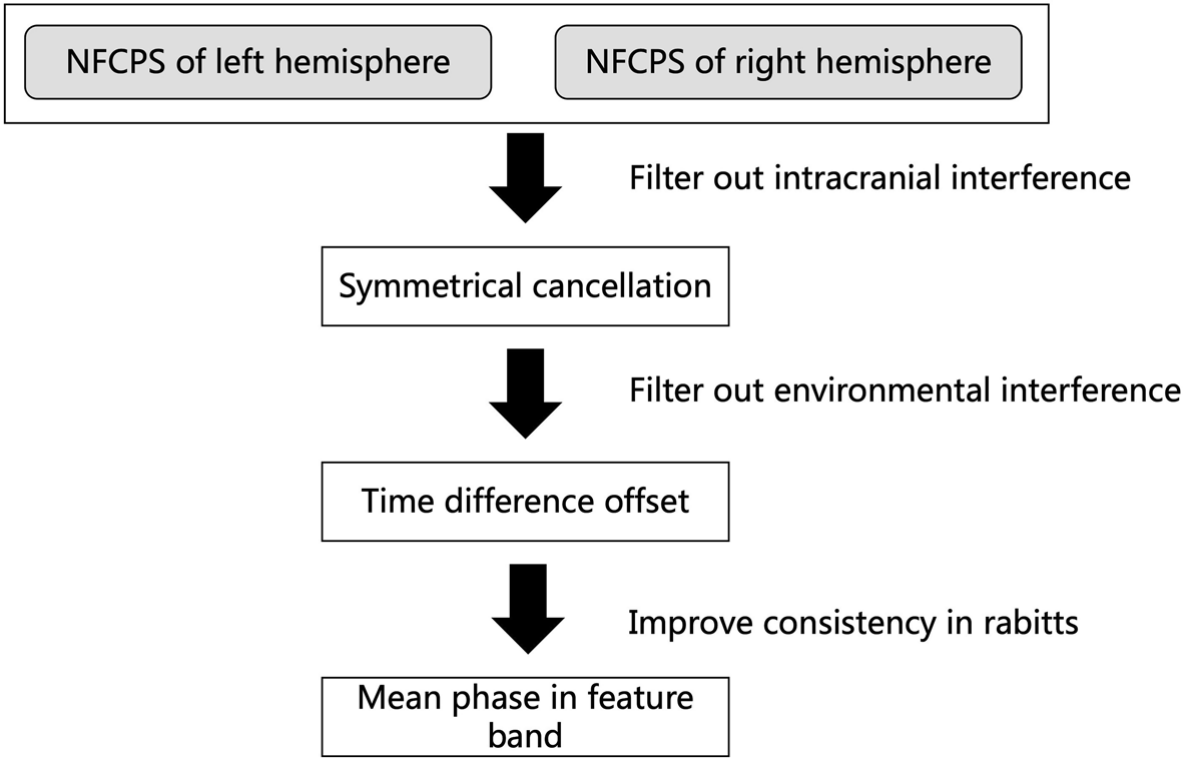
Processing flowchart of NFCPS data

Based on the 24-h NFCPS data collected by the symmetric cancellation NFCPS monitoring system, we performed the data processing as shown in Fig. 12.

To minimize the interference from other intracranial pathophysiological activities and the external environment, the NFCPS monitoring outcomes were processed by symmetric cancellation and time difference offset. After symmetric cancellation, the information about cerebral edema was strengthened. In time difference offset, the NFCPS of the anesthetized rabbit was used as the reference value, and the NFCPS monitoring results from 0 to 24 h after modeling were all subtracted from it. Subsequently, the distribution changes of NFCPS in the detection frequency band at different time points were observed. Based on the regulation mechanism of CE and VE on intracranial electrical conductivity, we analyzed the feasibility of using NFCPS changes to reflect the occurrence and development of cerebral edema. In addition, we calculated the mean variation of NFCPS under the feature band (83–87 MHz) near the central frequency, that is, the mean phase in the feature band (MPFB), and analyzed its 24-h variation trend. Its mathematical description is as following:

2 $$ {\text{MPFB}} = \frac{{\mathop \sum \nolimits_{i = 1}^{n} {\text{NFCPS}}_{fi} }}{n} $$

$$ \left\{ {f_{1} \ldots f_{n} } \right\} \in \left\{ {83\;{\text{MHz}} \ldots 87\;{\text{MHz}}} \right\}. $$

### Statistical analysis

To assess whether there is a significant difference in MPFB between the experimental group at the time point *η* (when CE transitions into VE) and the control group in a normal state, we determined *η* based on the trend of MPFB over time. Subsequently, we calculated the confidence intervals for these two groups at this moment, along with the *p*-value from an independent samples *t*-test. If the *p*-value is less than 0.05, it suggests a significant difference between the two groups. Moreover, we also compared the MPFB at the *η* moment and at the 24th hour to verify any significant difference. For this, we calculated the confidence interval for the MPFB at the 24th hour after the onset of cerebral edema, and compared this value with the MPFB of the experimental group at the *η* moment using a paired samples *t*-test. Again, if the *p*-value is less than 0.05, it can be concluded that there is a significant difference in MPFB values between these two time points.

### Identification of cerebral edema types

Features under the 1-h and 2-h time windows were extracted separately. Then, the classification model for CE and VE was constructed by the SVM. The performances under several feature combinations were compared.

For the 1-h time window, the MPFB at the current time (*t*_0_) was defined as *α*. In addition, the change amplitude of MPFB from *t*_0_ to the next hour (*t*_1_) was regarded as *β*. Its mathematical description was as following:

3 $$ \beta = \alpha_{{t_{1} }} - \alpha_{{t_{0} }} . $$

For the 2-h time window, the change amplitude of MPFB from *t*_0_ to the next 2 h (*t*_2_) was regarded as *γ*. And its corresponding change rate was defined as *δ*. Their mathematical descriptions were as following:

4 $$ \gamma = \alpha_{{t_{2} }} - \alpha_{{t_{1} }} , $$

5 $$ \delta = \frac{{\alpha_{{t_{2} }} - \alpha_{{t_{0} }} }}{2}. $$

Under the two time windows, we constructed different datasets based on 5 feature combinations (*α*, *β*, *αβ*, *αδ*, *αβγ*) and trained them separately. Supervised learning was adopted in this work, and a label set for CE and VE was added to every data set. Based on leave-out method, the training set, validation set and test set were randomly divided in a ratio of 6:2:2.

**Fig. 13:**
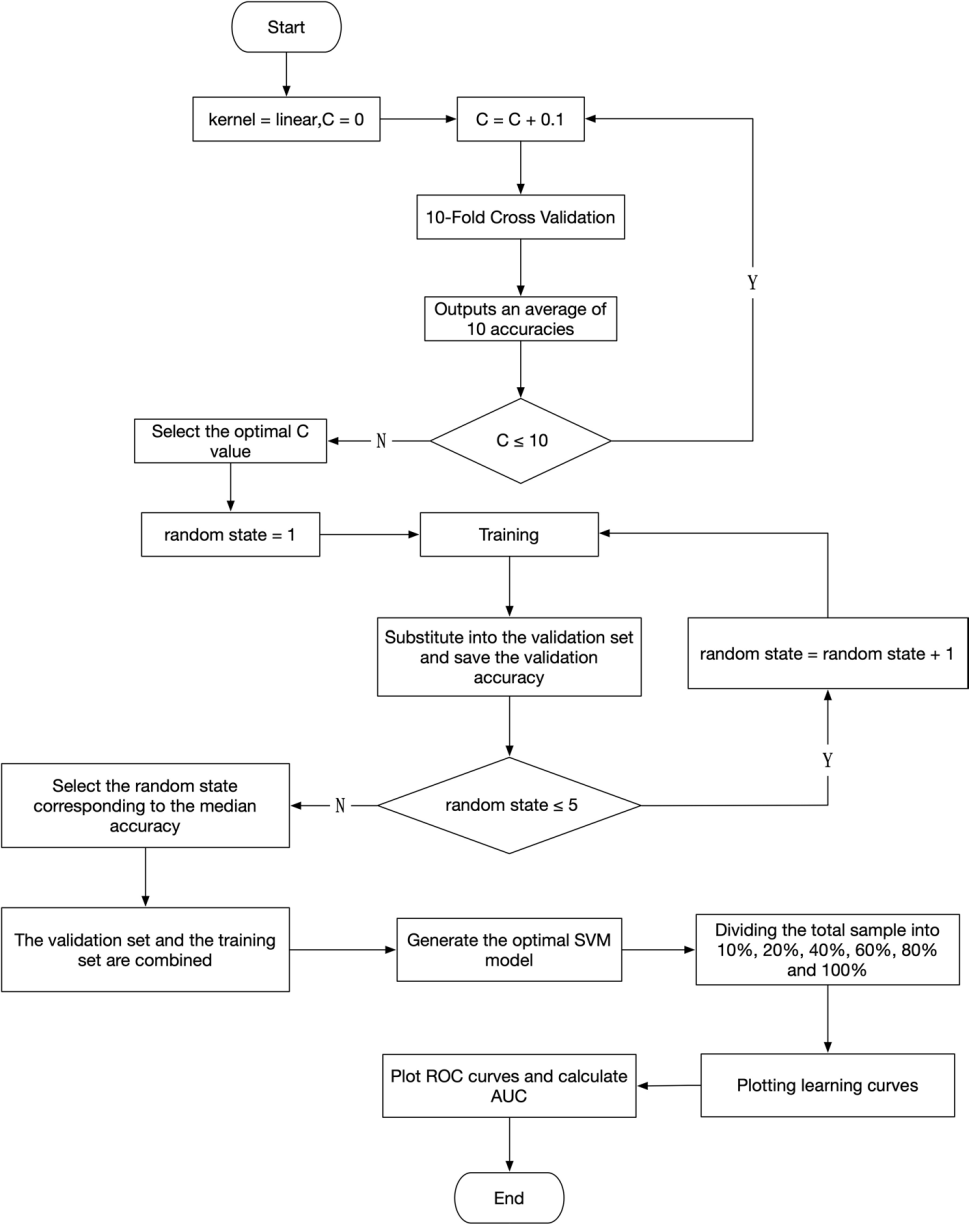
Construction and testing process of classification model for CE and VE

As shown in Fig. 13, SVM was selected to construct the identification model of cerebral edema types. When the linear kernel function was adopted, the mathematical expression of the SVM was as follows:

6 $$ \mathop {\max }\limits_{\alpha } \mathop \sum \limits_{i = 1}^{m} \alpha_{i} - \frac{1}{2}\mathop \sum \limits_{i = 1}^{m} \mathop \sum \limits_{j = 1}^{m} \alpha_{i} \alpha_{j} y_{i} y_{j} K\left( {x_{i} ,x_{j} } \right) $$

$$ {\text{s}}.{\text{t}}. \mathop \sum \limits_{i = 1}^{m} \alpha_{i} y_{i} = 0, $$

$$ \alpha_{i} \ge 0,i = 1,2, \ldots ,m. $$

7 $$ K\left( {x_{i} ,x_{j} } \right) = x_{i}^{T} x_{j} . $$

In practice, a penalty factor was generally added to construct a soft-margin SVM to obtain better generalization. Its mathematical expression was as follows:

8 $$ \mathop {\min }\limits_{{\omega ,b,\xi_{i} }} \frac{1}{2}||\omega ||^{2} + C\mathop \sum \limits_{i = 1}^{m} \xi_{i} , $$

$$ {\text{s}}.{\text{t}}. \;y_{i} \left( {\omega^{T} x_{i} + b} \right) \ge 1 - \xi_{i} , $$

$$ \xi_{i} \ge 0,i = 1,2, \ldots ,m. $$

$C$ is the penalty factor. $\xi_{i}$ represents the slack variable, reflecting the classification loss of one sample. $\mathop \sum \limits_{i = 1}^{m} \xi_{i}$ reflects the total error. The $C$ was increased by 0.1 in a loop (*n* = 100), and the average accuracy was output based on tenfold cross-validation (Additional file 8). Then, the lowest $C$ with the highest average accuracy was used for the classification model. Parameter optimization of the model was performed by adding random states. First, the random states numbered 1 to 5 were selected for training, respectively. Then, they were substituted into the validation set to output the accuracy. And the random state for the data set was determined based on the median of the 5 accuracy. Finally, the validation set and the training set were performed combine training to obtain the optimal model.

The overfitting and underfitting of the constructed model were analyzed by learning curves. (i) *Overfitting*. With the increase of training samples, the training error was increasing and the test error was decreasing, but the training error was much lower than the test error. (ii) *Underfitting*. When training samples were enough, both the training error and the test error were high, and the training error was approximately equal to the test error.

To investigate the performance of the constructed model, we compared and analyzed the classification accuracy of several feature combinations (*α*, *β*, *αβ*, *αδ*, *αβγ*), respectively. In addition, the specificity was assessed by receiver operating characteristic (ROC) curve and the area under the curve (AUC).

### Supplementary Information

**Additional file 1.** All data

**Additional file 2.** α data

**Additional file 3.** β data

**Additional file 4.** αβ data

**Additional file 5.** SVM code

**Additional file 6.** αδ data

**Additional file 7.** αβγ data

**Additional file 8.** Tenfold cross-validation result

## Data Availability

The datasets used and/or analyzed during the current study are available from the corresponding author on reasonable request. All data generated or analysed during this study are included in this published article.
