# Correction: Point-of-care testing for cerebral edema types based on symmetric cancellation near-field coupling phase shift and support vector machine

**DOI:** 10.1186/s12938-023-01162-3

**Published:** 2023-10-17

**Authors:** Mingyan Li, Rui Zhu, Gen Li, Shengtong Yin, Lingxi Zeng, Zelin Bai, Jingbo Chen, Bin Jiang, Lihong Li, Yu Wu

**Affiliations:** 1 School of Pharmacy and Bioengineering, Chongqing University of Technology, Chongqing, 400054 China; 2 College of Artificial Intelligence, Chongqing University of Technology, Chongqing, 401135 China; 3 Department of Neurosurgery, Southwest Hospital, Army Medical University, Chongqing, 400038 China; 4 College of Biomedical Engineering, Army Medical University, Chongqing, 400038 China

**Correction:** **BioMedical Engineering OnLine (2023) 22:80** **https://doi.org/10.1186/s12938-023-01145-4**

In this article [1], the funding section for this article should read as “This research was supported by the National Natural Science Foundation of China (No. 62001070). This work was also supported by the Natural Science Foundation of Chongqing (No. cstc2020jcyj-msxmX0322) and the Graduate Student Innovation Program of Chongqing University of Technology (No. gzlcx20222094 and No. gzlcx20232111). This work was also supported by the Chongqing University of Technology Research and Innovation Team Cultivation Program (No. 2023TDZ012). This work was supported by the Science and Technology Research Program of Chongqing Municipal Education Commission (KJQN202001122)”.

The original article has been corrected.
